# Wandering spleen presenting as a pelvic mass: A rare case report and literature review

**DOI:** 10.1016/j.radcr.2025.05.063

**Published:** 2025-06-20

**Authors:** Hajar Siouri, Hajar Betari, Amal Mojahid, Nadia El Mahi, Hamid Ziani, Nasri Siham, Imane Kamaoui, Imane Skiker

**Affiliations:** Department of Radiology, Mohammed VI University Hospital, Faculty of Medicine, University Mohammed First, Oujda, Morocco

**Keywords:** Ectopic spleen, Wandering spleen, Pelvic mass, Acute abdominal pain, Splenopexy, Splenectomy, Vascular pedicle

## Abstract

Wandering spleen is a rare condition caused by the absence or laxity of the ligaments that normally hold the spleen in place, resulting in its abnormal mobility within the abdominal or pelvic cavity. We report the case of a 15-year-old girl who presented with acute abdominal pain and nonspecific clinical findings. Abdominal CT revealed an enlarged ectopic spleen in the pelvic region with a long, tortuous vascular pedicle, but no signs of torsion or infarction. This case highlights the variable clinical presentation of wandering spleen, which can range from incidental discovery to acute abdomen, with imaging particularly CT being crucial for diagnosis. In uncomplicated cases, splenopexy is preferred to preserve splenic function, whereas splenectomy is reserved for infarcted or nonviable spleens. The case underscores the importance of considering wandering spleen in adolescents with unexplained abdominal pain and demonstrates how early diagnosis and conservative surgical management can prevent serious complications.

## Introduction

The spleen is an intraperitoneal organ located in the left hypochondrium behind the stomach, measuring approximately 12 cm in length and 7 cm in width. It is typically fixed in the left upper quadrant by the lienogastric, lienorenal, and phrenicocolic ligaments. However, in cases where there is incomplete fusion of the posterior mesogastrium, the spleen may become mobile [1,2]. An ectopic spleen is a rare condition in which the spleen exhibits abnormal mobility, leading it to be located outside its usual position. The exact cause of this condition remains unknown, but several factors contribute to its development. It typically occurs due to the laxity or absence of the ligaments that normally secure the spleen in the left upper quadrant, allowing it to shift to different parts of the abdomen depending on the length of the elongated vascular pedicle [[2], [3], [4]]. An ectopic spleen can be congenital, resulting from the absence of the gastrosplenic and splenorenal ligaments, or acquired, due to weakening of the ligaments that support the spleen [5,6]. These abnormalities result in a long vascular pedicle, making the spleen hypermobile and more prone to torsion. Ectopic spleen is a rare condition, with an incidence of less than 0.5% [2,7], and is more frequently observed in women aged 20-40 years [8,9] and children under 10 years old [8,9]. While it is uncommon for the spleen to be found outside its usual location in the left upper quadrant [4], its presence in the pelvis is particularly rare [7]. Due to the long vascular pedicle, the spleen can be located anywhere in the abdomen or pelvis.

Patients with an ectopic spleen may be asymptomatic and discovered incidentally during imaging studies, or they may present with abdominal pain or a palpable mass without additional symptoms. Imaging is crucial for diagnosis, with computed tomography helping to identify the mass. Treatment is determined based on the viability of the spleen, with splenopexy being the preferred option, except in cases of infarction, where splenectomy may be necessary.

## Case report

A 15-year-old female with no known medical or surgical history presented to the emergency department with a 3-day history of acute abdominal pain. She recalled a similar episode of abdominal pain approximately 1 year earlier, which resolved spontaneously without medical evaluation. There was no significant family medical history reported.

On physical examination, the patient had abdominal distension with localized tenderness in the lower abdomen. A painful, palpable mass was noted in the same region. Her vital signs on admission were stable: blood pressure was 110/70 mmHg, respiratory rate 22 breaths per minute, heart rate 85 beats per minute, temperature 37.5 °C, and oxygen saturation (SpO₂) was 97%. Laboratory investigations revealed normal values: white blood cell count of 5000/mm³ (reference range: 4000-11,000/mm³), hemoglobin level of 12.8 g/dL (reference range: 11.5-16.5 g/dL), and blood type A positive.

Abdominal ultrasonography initially identified a mass in the lower abdomen. A subsequent computed tomography (CT) scan confirmed the absence of the spleen in its usual anatomical location and revealed an enlarged ectopic spleen measuring 17 cm in length, located in the right lower abdomen and extending into the pelvic cavity. The spleen was in close contact with the bladder and uterus. The splenic pedicle appeared twisted and dilated, consistent with the “whirl sign,” although splenic vascularization was preserved, ruling out ischemia.

Given the significant risk of complications related to torsion of the splenic pedicle and despite the preserved vascularization observed on imaging, the patient was scheduled for a splenectomy to prevent any recurrence or worsening of the condition. The surgical intervention was planned urgently to remove the ectopic spleen responsible for the acute symptoms.

## Discussion

The spleen is a highly vascularized intraperitoneal organ involved in both immune defense and hematopoietic functions, typically located in the left upper quadrant of the abdomen. Its anatomical stability is ensured by peritoneal ligaments, primarily the gastrosplenic ligament—linking it to the greater curvature of the stomach—and the splenorenal ligament—attaching it to the left kidney and the posterior abdominal wall. The splenic hilum, positioned anteromedially, contains the splenic artery and typically 6 or more branches of the splenic vein. Splenic size is variable, depending on both age and body weight [[1], [2], [3]]. An ectopic spleen, also known as a wandering spleen, is an uncommon clinical condition in which the spleen is abnormally displaced from its usual anatomical location. This condition has a reported prevalence of less than 0.5% in the general population and is most often diagnosed in children and in women aged 20 to 40 years [8,9].

The etiology of ectopic spleen is multifactorial, but it is most commonly attributed to congenital anomalies involving the dorsal mesogastrium, leading to absence, malformation, or laxity of the splenic suspensory ligaments [1,2,9,8]. These ligaments—namely the gastrosplenic, splenorenal, and splenocolic ligaments—are crucial in maintaining the spleen’s position. Additional ligaments, such as the splenoomental and splenophrenic, may be present variably. In their absence or underdevelopment, gravitational force allows the spleen to descend into the lower abdomen, either on the left or right side, suspended solely by an elongated vascular pedicle [6,7]. Ectopic spleen may be incidentally discovered or become symptomatic, often presenting with nonspecific abdominal discomfort resulting from torsion of the vascular pedicle. While many patients are asymptomatic, some may present with complications including torsion, hemorrhage, spontaneous rupture, or the formation of cysts. The clinical presentation ranges from mild, intermittent symptoms to acute abdominal emergencies. Torsion of the splenic pedicle is the most frequently reported complication and a common cause of symptoms [7,9]. When the splenic vessels become twisted, vascular congestion may occur, leading to splenomegaly, which is regarded as a result rather than a cause of the ectopic positioning [6,7]. On physical examination, the most typical finding is a palpable abdominal mass, usually in the lower quadrants, corresponding to the displaced and possibly enlarged spleen. Although laboratory findings are often nonspecific, they may occasionally reveal signs of hypersplenism or functional asplenia. Approximately 60% of ectopic spleen cases present with abdominal pain or a mass, while the remaining 40% are asymptomatic [8].

Ultrasonography, computed tomography (CT), and magnetic resonance imaging (MRI) are the most reliable diagnostic tools, enabling accurate localization of the spleen and assessment of vascular integrity. Among these, ultrasound and CT are the most commonly used in clinical practice. Early diagnosis is critical to avoid serious complications, such as splenic infarction, secondary to torsion of the vascular pedicle.

Splenopexy is the preferred treatment for ectopic spleen, particularly in the absence of infarction, as it allows preservation of splenic function. Splenectomy is reserved for cases with infarction or irreversible vascular compromise [4], which can typically be confirmed via imaging. According to the literature, splenectomy for ectopic spleen accounts for less than 0.25% of all splenectomies [1,4,5]. When performed appropriately, splenopexy has been shown to be effective in preventing complications and maintaining immunological splenic function [1,4].

## Conclusion

Ectopic spleen is a rare but clinically significant condition, most often resulting from congenital anomalies or laxity of the splenic suspensory ligaments, allowing abnormal migration of the spleen within the abdominal or pelvic cavity. Its diagnosis relies heavily on imaging, with abdominal computed tomography (CT) being the gold standard. CT scans enable precise evaluation of the spleen’s position, size, and vascular integrity. Key radiological features include the absence of the spleen from its normal location in the left upper quadrant and the presence of a mobile, well-enhancing mass resembling normal splenic tissue elsewhere in the abdomen or pelvis. The choice of treatment depends on spleen viability. Splenopexy is the preferred option when the spleen is viable, as it effectively prevents complications while preserving immunologic function. Splenectomy is indicated only in cases of splenic infarction or irreversible torsion, both of which can be accurately diagnosed through imaging.

**Fig. 1:**
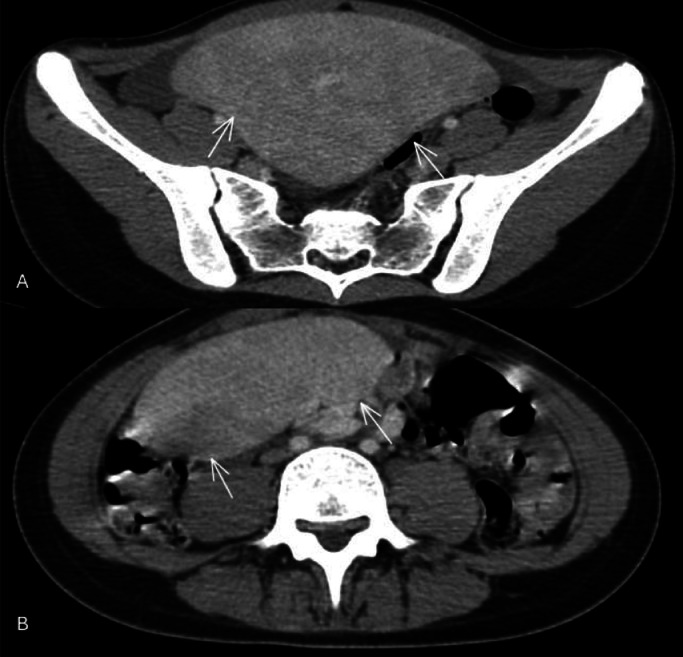
Axial (A) and (B) reformatted CT scan images of the abdomen post IV contrast administration, showing an ectopic spleen (white arrows) in the pelvic region.

**Fig. 2:**
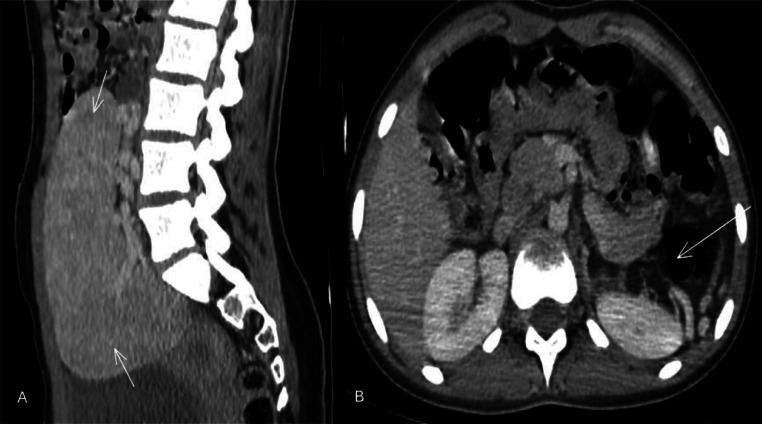
Contrast-enhanced axial and sagittal CT images of the upper abdomen showing the absence of splenic tissue in the left upper quadrant, with an enlarged ectopic spleen located in the pelvic region and a mild delay in homogenization during the portal phase.

**Fig. 3:**
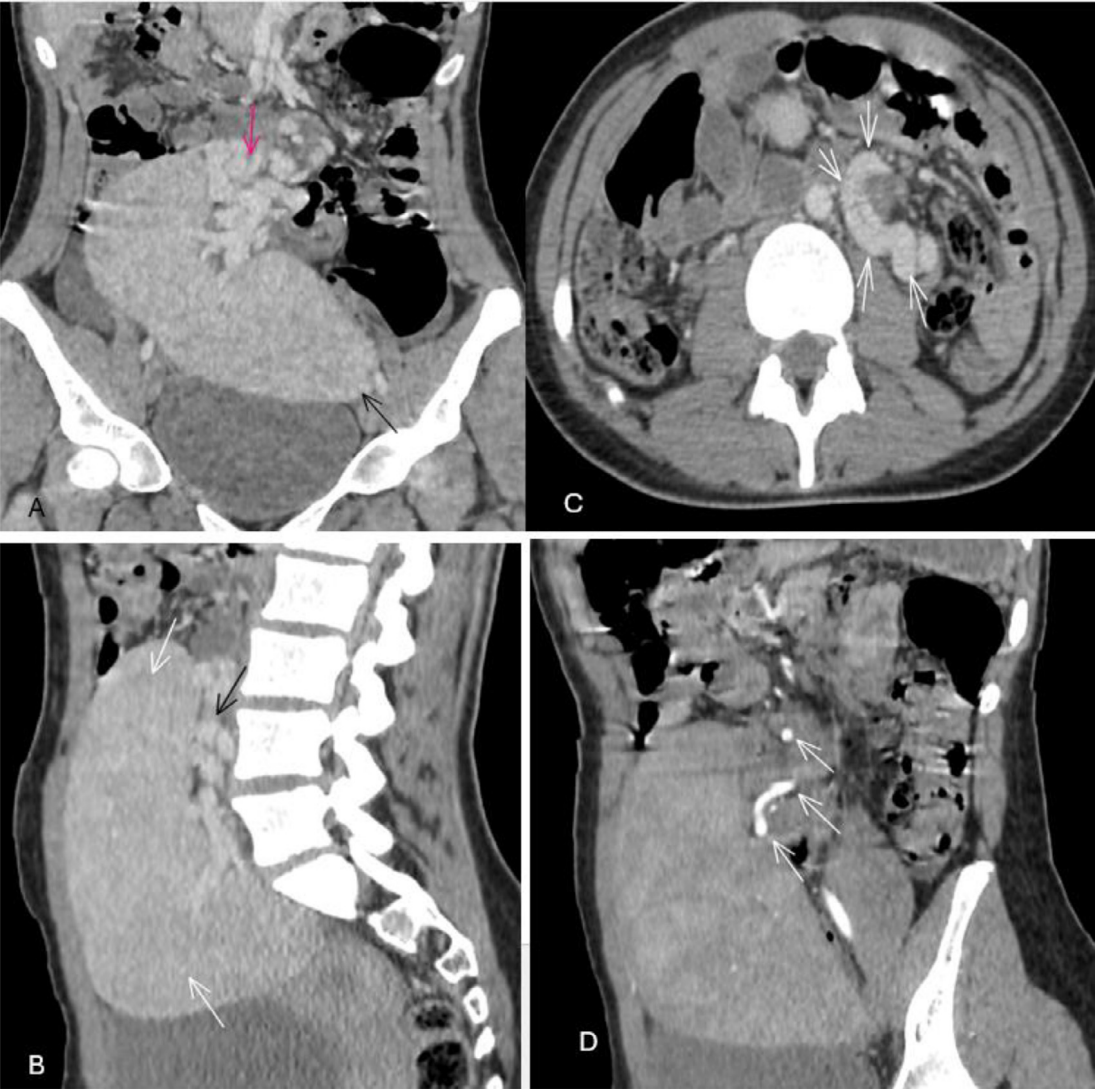
Post-IV abdominal CT images: venous phase (A-C) and arterial phase (D) showing an ectopic spleen located in the pelvic region. A long tortuous vascular pedicle containing the splenic vessels (C, white arrows: whirl sign) is seen extending from the epigastric region to the prolapsed spleen. There was a long splenic pedicle containing tortuous vessels with the splenic vein and artery. Sagittal contrast enhanced CT image showing a large ectopic spleen in the pelvis suspended by an elongated and tortuous vascular pedicle.

Early diagnosis and appropriate surgical management are essential to prevent potentially life-threatening complications and to ensure optimal patient outcomes (Fig. 1, Fig. 2, Fig. 3).

## Patient consent

An informed consent was obtained from the patient.

## References

[1] Qazi S.A., Mirza S.M., Muhammad A.M., Al Arrawi M.H., Al-Suhaibani Y.A. (2004). Wandering spleen. Saudi J Gastroenterol.

[2] Vlastarakos P., Rouvali A., Giourga M., Gerede A., Domali E. (2021). Wandering spleen: a rare case of an adnexal lesion. Cureus.

[3] Kumar B., Agrawal A., Gupta V., Agrawal V., Goyal S. (2016). Wandering spleen: a rare diagnosis with variable presentation. Radiol Case Rep.

[4] Satyadas T., Nasir N., Bradpiece HA. (2002). Wandering spleen: case report and literature review. J R Coll Surg Edinb.

[5] Lim Y., Leng H., Lee C.H., Chhun V., Lee YD. (2022). A congenital wandering spleen with a large epithelial cyst: a case report. Clin Case Rep..

[6] Desai D.C., Hebra A., Davidoff A.M., Schnaufer L. (1997). Wandering spleen: a challenging diagnosis. South Med J.

[7] Zarrintan S., Jamali F., Tubbs R.S., Khaki A.A., Salavati A., Tanoomand A. (2007). A wandering spleen presenting as a pelvic mass: case report and review of the literature. Folia Morphol (Warsz).

[8] Al-Hassani A., Al-Kuwari M., Al-Sulaiti M., Al-Kuwari A. (2013). Wandering spleen with torsion of the pedicle: a case report. Saudi Med J.

[9] Misawa T., Yoshida K., Shiba H., Kobayashi S., Yanaga K. (2008). Wandering spleen with chronic torsion. Am J Surg.

